# Cellular MicroRNAs 498 and 320d Regulate Herpes Simplex Virus 1 Induction of Kaposi’s Sarcoma-Associated Herpesvirus Lytic Replication by Targeting RTA

**DOI:** 10.1371/journal.pone.0055832

**Published:** 2013-02-13

**Authors:** Qin Yan, Wan Li, Qiao Tang, Shuihong Yao, Zhigang Lv, Ninghan Feng, Xinting Ma, Zhiqiang Bai, Yi Zeng, Di Qin, Chun Lu

**Affiliations:** 1 State Key Laboratory of Reproductive Medicine, Nanjing Medical University, Nanjing, People’s Republic of China; 2 Key Laboratory of Pathogen Biology of Jiangsu Province, Nanjing Medical University, Nanjing, People’s Republic of China; 3 Department of Microbiology and Immunology, Nanjing Medical University, Nanjing, People’s Republic of China; 4 Department of Clinical Laboratory, The Affiliated Nanjing First Hospital of Nanjing Medical University, Nanjing, People’s Republic of China; 5 Department of Medicine, Quzhou College, Quzhou, People's Republic of China; 6 Department of Clinical Laboratory, Jiangsu Province Official Hospital, Nanjing, People’s Republic of China; 7 Department of Urology, The First Affiliated Hospital of Nanjing Medical University, Nanjing, People’s Republic of China; 8 Department of Microbiology and Immunology, Youjiang Medical College for Nationalities, Bose, People’s Republic of China; University of Nebraska - Lincoln, United States of America

## Abstract

Kaposi’s sarcoma-associated herpesvirus (KSHV) infection was necessary but not sufficient for KS development without other cofactors. We have previously reported that herpes simplex virus (HSV)-1 was an important cofactor that reactivated KSHV from latency by inducing the expression of KSHV replication and transcription activator (RTA), the lytic switch protein. Here, we further investigated the possible cellular microRNAs (miRNAs) involved in regulation of RTA during HSV-1-induced KSHV replication. The differential profiles of miRNAs expression between Mock- and HSV-1-infected body cavity-based lymphoma (BCBL-1) cells were identified by miRNA microarray analysis. Bioinformatics and luciferase reporter analyses showed that two of the HSV-1-downregulated cellular miRNAs, miR-498 and miR-320d, directly targeted the 3′ untranslated region (UTR) of KSHV RTA. As a result, overexpression of these two miRNAs significantly inhibited HSV-1-induced KSHV replication, whereas repression of these miRNAs with specific suppressors enhanced HSV-1-mediated KSHV replication. In addition, miR-498 or miR-320d alone, without HSV-1 infection, regulated KSHV replication in BCBL-1 cells. Finally, bioinformatics Gene Ontology (GO) analysis indicated that targets of HSV-1-regulated miRNAs were enriched for proteins, whose roles were involved in protein binding, enzyme activity, biological regulation, and several potential signaling pathways including transforming growth factor (TGF)-β were likely to participate in HSV-1-induced KSHV replication. Collectively, these novel findings demonstrated that host-encoded miR-498 and miR-320d regulated HSV-1 induction of KSHV lytic replication by targeting RTA, which provided further insights into the molecular mechanisms controlling KSHV lytic replication.

## Introduction

Kaposi’s sarcoma-associated herpesvirus (KSHV), also called human herpesvirus (HHV)-8, was first identified in 1994 [1]. KSHV is the etiological agent of KS, a complex neoplasm induced by KSHV-infected endothelial cells. KSHV is also strongly associated with the B-cell proliferative disorder primary effusion lymphoma [PEL, also termed body-cavity-based lymphoma (BCBL)] and some cases of multicentric Castleman’s disease [2]. Like all herpesviruses, KSHV displays two distinct life stages, latency and lytic replication. During latency, KSHV expresses highly restricted proteins, thus limiting immune exposure while allowing persistence of the virus. Once KSHV is reactivated from latency and enters the lytic cycle, most viral genes are expressed in an orderly fashion [immediate-early (IE), early and late], leading to the production of infectious virions [2]. Both latent and lytic genes have a role in KSHV pathogenesis, and the balance between latency and lytic replication contributes to KS development.

Although KSHV infection appears to be necessary but not sufficient for the development of KS, other cofactors play an important role. We and others have demonstrated that several agents, such as human immunodeficiency virus (HIV)-1, human cytomegalovirus (HCMV), HHV-6 and herpes simplex virus (HSV)-2, have been proved to be cofactors that reactivate KSHV from latency [3]–[8]. Progression through the lytic cycle and replication of the viral genome are of unique importance because lytic reactivation of KSHV is an essential pathogenic step. We have also shown that HSV-1 is another important cofactor that reactivates lytic replication of KSHV [9], [10]. HSV-1 is a ubiquitous virus that infects the majority of human population (approximately 60–90%) [11]. Coinfection of HSV-1 and KSHV were frequently detected in acquired immunodeficiency syndrome (AIDS) or KS patients, recurrent aphthous ulceration patients, and even in healthy individuals [12]–[16]. Although HSV-1 and KSHV are not found in similar anatomic compartments during their latent infection, frequent reactivation of latent HSV-1 occurred in AIDS or AIDS-KS patients, leading to appearance of HSV-1 viraemia [17]. Viraemia is not only present in immunocompromised individuals, but also in immunocompetent individuals [18]–[21]. HSV-1 viraemia subsequently increased opportunities for HSV-1 to contact B and/or endothelial cells [HSV-1 could infect B cells and human vascular endothelial cells (the precursor of KS)] [22]–[27], which maybe previously had harboured KSHV genome. Indeed, we have proved that HSV-1 infected BCBL-1 cells and reactivated KSHV from latency through induction of KSHV replication and transcription activator (RTA) [9], [10]. It is noteworthy that RTA (also known as ORF50) of KSHV is a molecular switch that initiates productive replication of latent KSHV genomes [28]. RTA is the IE protein of KSHV and it transactivates a number of viral genes and autoregulates its own expression via various DNA elements within promoter regions. RTA is necessary and sufficient to switch latent KSHV into the lytic infection cycle. However, the specific molecular mechanisms of induction of KSHV RTA expression by HSV-1 are not well known. Although we have found that HSV-1 infection promoted the induction of KSHV RTA promoter activity [9], it is not known that whether HSV-1 activates KSHV replication by regulating some important microRNAs (miRNAs) that target RTA or other key genes of KSHV.

Recently, the discovery of miRNAs in herpesviruses has added another layer of complexity to our understanding of KSHV pathogenesis [29]. MiRNAs are about 22 nucleotide (nt) noncoding RNAs that act by binding mainly to the 3′-untranslated region (UTR) of specific mRNAs, targeting them for degradation or translation repression [30]. KSHV produces 25 mature miRNAs from 12 pre-miRNAs, all of which are encoded in the latency locus [31]. The mature KSHV miRNAs are called miR-K12-1-12, based on their proximity to the Kaposin (K12) gene, or simply miR-K1-12. Recent studies have demonstrated that KSHV miRNAs are likely to play key roles in the viral life cycle, immune evasion, and transformation of host cells by directly regulating viral genes or indirectly regulating host genes [32]–[39]. It is known that cellular miRNAs are linked to almost all known cellular processes, including proliferation, differentiation, and apoptosis. Increasing evidence suggests that host miRNAs not only participate in maintenance of normal cell functions, but are also involved in host–virus interactions and play a key role in the regulation of viral replication [40]–[46]. With regard to host miRNAs and KSHV replication, it has been shown that miR-132 is highly upregulated after KSHV infection of primary human lymphatic endothelial cells (LECs) and has a negative effect on the expression of interferon-stimulated genes, facilitating KSHV replication [47]. Thus, whether cellular miRNAs play roles in HSV-1-induced KSHV replication is not known.

To clarify the molecular mechanisms of KSHV activation by HSV-1 from the perspective of miRNAs, in this study, we identified two cellular miRNAs (miR-498 and miR-320d) that regulated HSV-1-induced KSHV replication, by targeting KSHV lytic switch protein RTA. This is believed to be the first study to report the involvement of cellular miRNAs in the induction of lytic replication of KSHV by HSV-1, which implies their important role in the pathogenesis of KSHV-associated malignancies.

## Results

### Screening and Validation of MiR-498 and MiR-320d that Target KSHV RTA 3′UTR

**Figure 1:**
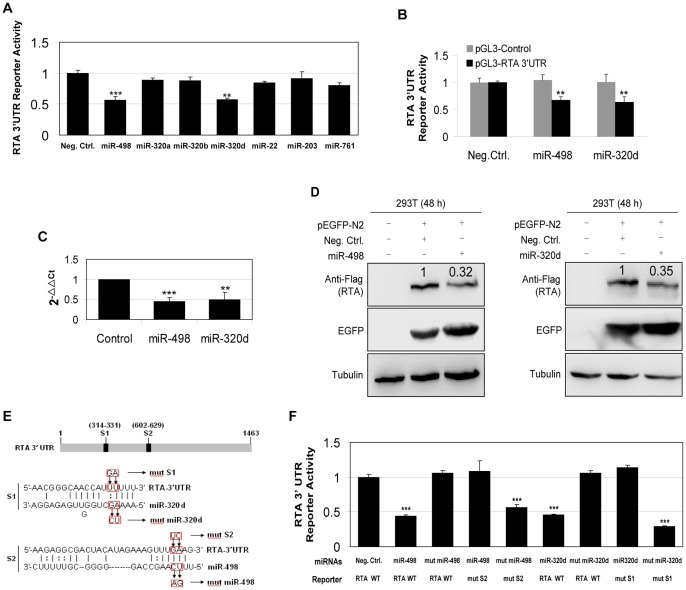
HSV-1 infection of BCBL-1 cells downregulates miR-498 and miR-320d, both of which directly target KSHV RTA. (**A**)**. Luciferase reporter assay for screening miRNAs that target KSHV RTA 3′UTR.** 293T cells were co-transfected with negative control nucleotide of miRNA (**Neg. Ctrl.**) or mimics of several miRNAs together with the pGL3-Luc-RTA 3′UTR luciferase reporter and assayed for luciferase activity. **P<0.01 and ***P<0.001 for Student’s t-test versus Neg. Ctrl. group. (**B**)**. Both miR-498 and miR-320d only inhibited the reporter activity of pGL3-RTA 3′UTR but not that of pGL3-Control construct.** Luciferase activity was detected by co-transfection pGL3-Control or pGL3-RTA 3′UTR construct along with Neg. Ctrl., mimic of miR-498 (**miR-498**) or miR-320d (**miR-320d**) for 24 h in 293T cells. The relative reporter activity levels of pGL3-RTA 3′UTR and pGL3-Control in the Neg. Ctrl. group were considered to be “1” for comparison, respectively. **P<0.01 for Student’s t-test versus pGL3-RTA 3′UTR plus Neg. Ctrl. group**.** (**C**)**. RT-qPCR analysis for validating the miRNA microarray data.** MiR-498 and miR-320d expression in BCBL-1 cells infected with HSV-1 or Mock for 24 h was quantitated by RT-qPCR. Relative quantities of miRNAs expression were represented as 2^−ΔΔCt^ on the y axis. **P<0.01 and ***P<0.001 for Student’s t-test versus Mock group. (**D**)**. Inhibition of RTA protein expression by miR-498 and miR-320d.** A genomic RTA expression vector pcDNA3.1−3×Flag-RTA-3′UTR bearing the full 3′UTR sequences was co-transfected with pEGFP and mimic of miR-498 or miR-320d into 293T cells for 48 h. Cells were collected and immunoblotted with the indicated antibodies. The relative level of RTA was determined by quantitative densitometry. Numbers labeled above the RTA band were the relative intensities of the bands compared to EGFP. The relative level of RTA in the Neg. Ctrl.+pcDNA3.1−3×Flag-RTA-3′UTR+pEGFP-N2 group was considered to be 1 for comparison. (**E**)**. Schematic illustration of the putative seed sequences of miR-320d** (**S1**) **and miR-498** (**S2**) **within the 3′UTR of RTA, and mutagenesis of target sites in the RTA 3′UTR or miRNA mimics.** Mutated nucleotides in the target sites were framed in red. (**F**)**. Effect of mutagenesis on miR-498 or miR-320d targeting of the 3′UTR of RTA.** After co-transfection of RTA wild type (**RTA WT**) or mutant 3′UTR construct (**mut S1 or mut S2**) together with natural (miR-498 or miR-320d) or mutant miR-498 or miR-320d mimic (mut miR-498 or mut miR-320d) for 24 h, 293T cells were assayed for luciferase activity. ***P<0.001 for Student’s t-test versus Neg.Ctrl.+RTA WT.

Recent studies have shown that KSHV-encoded miRNAs regulate viral latency and replication by targeting either cellular factors or KSHV key genes. To determine if cellular miRNAs were involved in HSV-1-induced KSHV replication, we examined the effect of HSV-1 infection of BCBL-1 cells on the expression of cellular miRNAs. As shown in Table 1, 109 miRNAs were downregulated and 75 miRNAs were upregulated after HSV-1 infection of BCBL-1 cells for 24 h. The lytic switch protein RTA is considered to be the master regulator of KSHV replication. Among 109 downregulated miRNAs, bioinformatics analysis identified 7 miRNAs that displayed the high potential to bind the 3′UTR of RTA. A luciferase reporter assay using RTA 3′UTR luciferase reporter (pGL3-Luc-RTA 3′UTR, which contained the full-length of KSHV RTA 3′UTR sequence downstream of the luciferase sequence in the pGL3-Promoter) confirmed that cellular miR-498 and miR-320d significantly decreased luciferase expression (Fig. 1A). However, we did not observe any synergistic or antagonistic effect of miR-498 and miR-320d on the RTA 3′UTR reporter activity (data not shown). Both miR-498 and miR-320d only inhibited the reporter activity of pGL3-RTA 3′UTR but not that of pGL3-Control construct (Fig. 1B). The expression of miR-498 and miR-320d by real-time quantitative polymerase chain reaction (RT-qPCR) was consistent with the miRNA microarray findings (Fig. 1C). Meanwhile, since our previous study have shown that HSV-1 infection of BCBL-1 cells increased KSHV gene expression as early as 3 h post-infection [9], we also detected the expression of miR-498 and miR-320d after HSV-1 infection of BCBL-1 cells for 0, 3, 6, and 12 h. The results from RT-qPCR revealed that the reduction of both miRNAs consonantly began with the dynamic change of KSHV gene expression (Fig. S1). In order to evaluate directly the effect of these two miRNAs on RTA expression, we performed transient co-transfection of the individual miRNA along with the plasmid pEGFP-N2 [pEGFP-N2 contained the enhanced green fluorescent protein (GFP) gene fused to the cytomegalovirus IE gene promoter] and RTA-expressing plasmid pcDNA3.1−3×Flag-RTA-3′UTR bearing the full 3′UTR sequences in 293T cells. Western blot indicated that both miRNAs strongly inhibited the expression of RTA, while the level of EGFP was measured for evaluation of the efficiency of transfection (Fig. 1D).

**Table 1:** MiRNAs differentially upregulated and downregulated >2 fold in BCBL-1 cells infected by HSV-1 for 24 h.

miRNA name	Fold	miRNA name	Fold	miRNA name	Fold	miRNA name	Fold	miRNA name	Fold
hsa-miR-502-5p	4.21	hsa-miR-138-1*	2.80	hsa-miR-4282	3.41	kshv-miR-K12-8	0.20	hsa-miR-3618	0.48
kshv-miR-K12-12*	2.50	hsa-miR-934	2.02	hsa-miR-24	0.42	hsa-miR-3679-3p	0.49	hsa-miRPlus-A1086	0.25
hsa-miR-1270	2.62	hsa-miR-3934	2.84	hsa-miR-501-5p	0.39	hsa-miR-205*	0.25	hsa-miR-1827	0.42
hsa-miR-3175	3.57	hsa-miR-3654	2.60	hsa-miR-92b	0.49	hsa-miR-210	0.39	hsa-miR-26a	0.44
hsa-miRPlus-I137*	2.27	hsa-miRPlus-J1011	2.08	kshv-miR-K12-12	0.08	hsa-miR-935	0.24	hsa-miR-628-3p	0.37
hsa-miR-3613-3p	2.68	hsa-miR-1280	2.08	hsa-miR-1246	0.32	hsa-miR-766	0.39	hsa-miR-99a	0.36
hsa-miR-933	4.93	hsa-miR-1301	2.59	hsv1-miR-H1	0.11	hsa-miR-4299	0.39	hsa-miR-320a	0.25
hsa-miR-335*	2.82	hsa-miR-138-2*	2.87	hsa-miR-29a	0.14	hsa-miR-3620	0.38	hsa-miR-22	0.15
hsa-miR-668	2.33	hsa-miR-3176	3.00	hsa-miR-423-5p	0.36	hsa-miR-203	0.13	kshv-miR-K12-5	0.48
hsa-miR-4325	6.96	hsa-miR-937	2.27	hsa-miR-125b	0.05	hsa-miR-1299	0.05	hsa-miR-106b	0.45
hsa-miR-593	6.71	hsa-miR-509-3-5p	3.06	hsa-miR-574-5p	0.44	hsa-miR-595	0.00	hsa-miR-1227	0.15
hsa-miR-183	2.24	hsa-miR-363*	3.15	hsa-miR-320c	0.30	hsa-miR-212	0.07	hsa-miR-7	0.21
hsa-miR-767-5p	2.80	hsa-miR-944	2.28	hsa-miR-424	0.11	hsa-miR-130b	0.18	hsa-miR-300	0.48
hsa-miR-2115*	2.36	hsa-miR-676*	6.46	hsa-miR-186*	0.08	hsa-miR-663b	0.12	hsa-miR-331-3p	0.39
hsa-miR-302c*	3.47	hsa-miR-3924	2.14	hsa-miR-658	0.15	hsa-miR-3687	0.04	hsa-miR-2115	0.17
hsa-miR-1205	6.78	hsa-miR-513a-3p	2.95	hsa-miR-4255	0.11	kshv-miR-K12-9*	0.43	hsa-miR-548t	0.41
hsa-miR-340	5.17	hsa-miR-375	3.61	hsa-miR-3651	0.49	hsa-miR-615-3p	0.31	hsa-miR-18b	0.11
hsa-miR-369-3p	3.94	hsa-miR-16	2.47	hsa-miR-320b	0.46	hsa-miR-377*	0.11	hsa-miR-3189	0.28
hsa-miR-548e	2.34	hsa-miR-1287	5.15	hsa-let-7a-2*	0.10	hsa-miR-9	0.48	hsa-miR-34a	0.11
hsa-miR-155	2.55	hsa-miR-3658	2.55	hsv1-miR-H5-3p	0.42	hsa-miRPlus-C1087	0.06	hsa-miR-3607-5p	0.33
hsa-miR-3649	2.63	hsa-miR-938	3.49	hsa-miR-92a	0.41	hsa-miR-2116	0,25	hsa-miR-181b	0.40
kshv-miR-K12-7	2.07	hsa-miR-451	33.89	hsa-miR-639	0.03	hsa-miR-1976	0.37	hsa-miR-373*	0.38
hsa-miR-486-5p	2.84	hsa-miR-487a	2.74	hsa-miR-4323	0.46	hsa-miR-224*	0.07	kshv-miR-K12-1	0.21
hsa-miR-559	3.28	hsa-miR-2113	2.31	hsa-miR-3667-5p	0.20	hsa-miR-625*	0.45	hsa-miR-181d	0.21
hsa-miR-145*	2.63	hsa-miR-4321	3.20	hsa-miR-941	0.48	hsa-miR-30e*	0.03	hsa-miR-20a	0.49
hsa-miR-208b	2.69	hsa-miR-4286	2.27	hsa-miR-193b*	0.40	hsa-miR-4308	0.32	hsa-miR-301a	0.31
hsa-miR-338-5p	3.61	hsa-miR-24-1*	2.09	hsa-miR-636	0.41	hsa-miR-1273e	0.11	hsa-miR-320d	0.42
hsa-miRPlus-C1110	2.55	hsa-miR-187*	2.05	kshv-miR-K12-11	0.10	hsa-miR-3655	0.25	hsa-miR-3653	0.23
hsa-miR-1913	2.43	hsa-miR-337-3p	2.22	hsa-miR-1297	0.09	hsa-miR-3675-3p	0.41	kshv-miR-K12-10a	0.45
hsa-miR-887	2.60	hsa-miR-1200	2.37	kshv-miR-K12-1*	0.13	hsa-miR-193a-3p	0.31	hsa-miR-574-3p	0.42
hsa-miR-299-3p	2.94	hsa-miR-1274a	2.39	hsa-miR-3607-3p	0.05	hsa-miR-186	0.08	hsa-miR-365	0.31
hsa-miR-589	3.13	hsa-miR-584	2.35	hsa-miR-3611	0.18	hsa-miR-25	0.31	hsa-miR-3171	0.03
hsa-miR-640	2.01	hsa-miR-600	2.08	hsa-miR-4278	0.14	hsa-miR-657	0.12	hsa-miR-3647-3p	0.34
hsa-miR-1274b	2.67	hsa-miR-323-3p	2.59	hsa-miR-654-5p	0.02	hsa-miR-556-3p	0.40	hsa-miR-761	0.45
hsa-miR-144	2312.36	hsa-miR-196b*	4.64	hsa-miR-575	0.25	hsa-miR-99b	0.22	hsa-miR-320e	0.30
hsa-miR-122*	2.20	hsv1-miR-H4*	2.06	hsa-miR-125a-5p	0.17	hsa-miR-498	0.40	hsa-miR-508-5p	0.46
hsa-miR-141*	4.18	hsa-miR-302e	2.16	hsa-miR-371-3p	0.03	kshv-miR-K12-8*	0.45		

The interaction of a miRNA with its target is primarily mediated by a 6–7-bp seed sequence at the 5′ end of the miRNA. Bioinformatics analysis identified one putative miR-498-binding site (S2) and one miR-320d-binding site (S1), respectively, in the RTA 3′UTR (Fig. 1E). Mutation of the putative binding sites from the RTA 3′UTR abolished the inhibitory effect of miR-498 or miR-320d on the RTA 3′UTR reporter activity (Fig. 1F). Mutant mimics without the seed sequences of miRNAs also failed to inhibit RTA 3′UTR reporter activity (Fig. 1F). These results indicate that both miR-498 and miR-320d directly target RTA 3′UTR.

### Overexpression of MiR-498 and MiR-320d Inhibits HSV-1-Induced KSHV Replication

**Figure 2:**
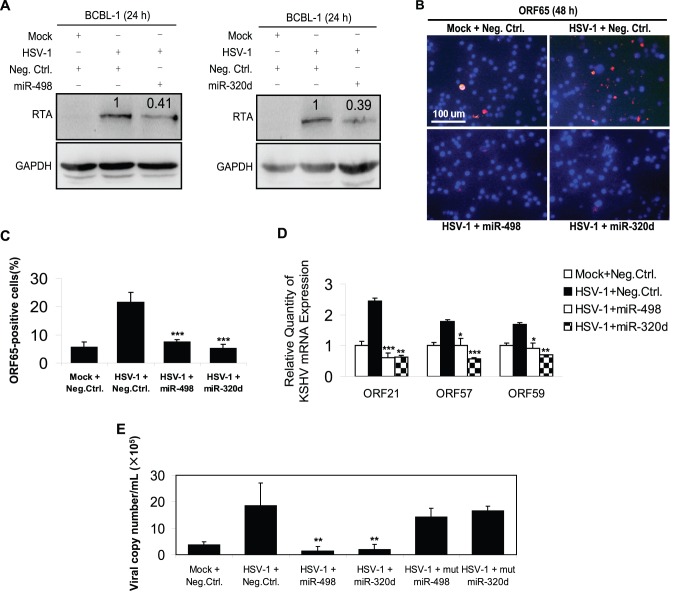
Overexpression of mimics of miR-498 and miR-320d inhibits HSV-1-induced KSHV lytic replication. (**A**)**. Transfection of miR-498 or miR-320d mimic inhibited HSV-1-induced KSHV RTA expression.** Western blot was used to detect the expression of RTA in BCBL-1 cells transfected with miR-498, miR-320d mimics or Neg. Ctrl. for 12 h, and infected with HSV-1 or Mock for another 24 h. The relative level of RTA was determined by quantitative densitometry. Numbers labeled above the RTA band were the relative intensities of the bands compared to GAPDH. The relative level of RTA in the HSV-1+ Neg. Ctrl. group was considered to be 1 for comparison. (**B**)**. Transfection of miR-498 or miR-320d mimic inhibited HSV-1-induced KSHV ORF65 expression.** KSHV lytic protein ORF65 expression was examined by IFA with ORF65 mAb in Neg. Ctrl. transfected and Mock infected (**Mock+Neg. Ctrl.**), Neg. Ctrl. transfected and HSV-1 infected (**HSV-1+ Neg. Ctrl.**), mimic of miR-498 transfected and HSV-1 infected (**HSV-1+ miR-498**) or mimic of miR-320d transfected and HSV-1 infected (**HSV-1+ miR-320d**) BCBL-1 cells (Original magnifications, ×10). (**C**)**. Quantification of results in B.** ***P<0.001 for Student’s t-test versus HSV-1+ Neg. Ctrl. group. (**D**)**. Transfection of miR-498 or miR-320d mimic inhibited HSV-1-induced KSHV ORF21/57/59 mRNA expression.** ORF21, ORF57 and ORF59 mRNA in BCBL-1 cells treated as in B was quantitated by RT-qPCR. *P<0.05, **P<0.01 and ***P<0.001 for Student’s t-test versus HSV-1+ Neg. Ctrl. group. (**E**)**. Transfection of miR-498 or miR-320d mimic inhibited HSV-1-induced KSHV progeny virions release.** Real-time DNA-PCR was used to detect the viral genome copy number in the supernatant of Neg. Ctrl. transfected and Mock infected (**Mock+Neg. Ctrl.**), Neg. Ctrl. transfected and HSV-1 infected (**HSV-1+ Neg. Ctrl.**), mimic of miR-498 transfected and HSV-1 infected (**HSV-1+ miR-498**), mimic of miR-320d transfected and HSV-1 infected (**HSV-1+ miR-320d**), mutant mimic of miR-498 transfected and HSV-1 infected (**HSV-1+ mut miR-498**), or mutant mimic of miR-320d transfected and HSV-1 infected (**HSV-1+ mut miR-320d**) BCBL-1 cells. **P<0.01 for Student’s t-test versus HSV-1+ Neg. Ctrl. group.

Western blot was performed to confirm whether both miRNAs could inhibit the expression of RTA in HSV-1-infected BCBL-1 cells. Transfection of miR-498 or miR-320d mimic significantly inhibited HSV-1-induced KSHV RTA expression (Fig. 2A) and increased their own expression without a doubt (Fig. S2). To explore whether both miRNAs were indeed involved in HSV-1-induced KSHV lytic replication by targeting RTA, we performed an immunofluorescence assay (IFA). As shown in Fig. 2B and 2C, the positive staining for KSHV minor capsid protein ORF65 was 2.92- and 4.17-fold lower in HSV-1-infected BCBL-1 cells transfected with mimic of miR-498 or miR-320d, respectively, than in cells transfected with control nucleotide. Similarly, the results from RT-qPCR demonstrated that both miRNAs decreased the expression of three KSHV lytic transcripts, ORF21, ORF57 and ORF59 (Fig. 2D). To determine further whether overexpression of miR-498 or miR-320d could reduce the release of KSHV progeny virions induced by HSV-1, experiments were designed to detect the copy number of KSHV progeny virions. The results of real-time DNA-PCR indicated that miR-498 or miR-320d mimic decreased KSHV genome copy number in the supernatant from HSV-1-infected BCBL-1 cells by 15.96- or 14.54-fold, respectively, while the mutant mimics failed to inhibit the release of KSHV progeny virions induced by HSV-1 (Fig. 2E). In addition, to determine whether the inhibition of miR-498 and miR-320d on HSV-1-induced KSHV replication was cell type specific, the same experiment was performed in another KSHV-positive PEL cell line, BC-3, which showed similar results to those in BCBL-1 cells (Fig. S3). These data collectively suggest that overexpression of miR-498 and miR-320d inhibits KSHV lytic replication induced by HSV-1.

### Repression of MiR-498 and MiR-320d Enhances HSV-1-Induced KSHV Replication

**Figure 3:**
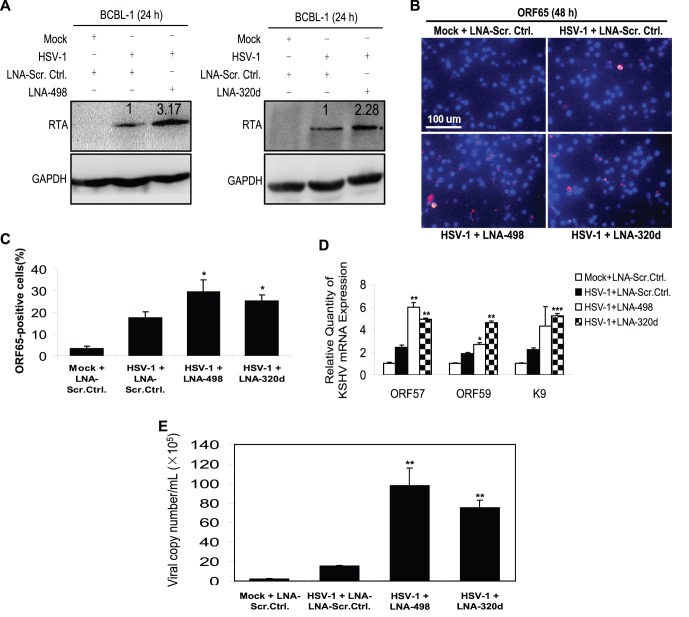
Inhibition of miR-498 and miR-320d promotes HSV-1-induced KSHV lytic replication. (**A**). Transfection of LNA-498 or LNA-320d enhanced HSV-1-induced KSHV RTA expression. Western blot was used to detect the expression of RTA in BCBL-1 cells transfected with LNA-498, LNA-320d or LNA scrambled control (**LNA-Scr. Ctrl.**) for 12 h, and infected with HSV-1 or Mock for another 24 h. The relative level of RTA was determined by quantitative densitometry. Numbers labeled above the RTA band were the relative intensities of the bands compared to GAPDH. The relative level of RTA in the HSV-1+ LNA-Scr. Ctrl. group was considered to be 1 for comparison. (**B**)**. Transfection of LNA-498 or LNA-320d enhanced HSV-1-induced KSHV ORF65 expression.** KSHV lytic protein ORF65 expression was detected by IFA with ORF65 mAb in LNA-Scr. Ctrl. transfected and Mock infected (**Mock+LNA-Scr. Ctrl.**), LNA-Scr. Ctrl. transfected and HSV-1 infected (**HSV-1+ LNA-Scr. Ctrl.**), LNA miR-498 transfected and HSV-1 infected (**HSV-1+ LNA-498**) or LNA miR-320d transfected and HSV-1 infected (**HSV-1+ LNA-320d**) BCBL-1 cells (Original magnifications, ×10). (**C**)**. Quantification of results in B.** *P<0.05 for Student’s t-test versus HSV-1+ LNA-Scr. Ctrl. group**.** (**D**)**. Transfection of LNA-498 or LNA-320d enhanced HSV-1-induced KSHV ORF57/59, K9 mRNA expression.** ORF57, ORF59 and K9 mRNA in BCBL-1 cells treated as in B was quantitated by RT-qPCR. *P<0.05, **P<0.01 and ***P<0.001 for Student’s t-test versus HSV-1+ LNA-Scr. Ctrl. group**.** (**E**)**. Transfection of LNA-498 or LNA-320d enhanced HSV-1-induced KSHV progeny virions release.** Real-time DNA-PCR was used to detect the viral genome copy number in the supernatant of BCBL-1 cells treated as in B. **P<0.01 for Student’s t-test versus HSV-1+ LNA-Scr. Ctrl. group.

Overexpression of these two miRNAs significantly inhibited HSV-1-induced KSHV replication; therefore, we aimed to establish whether repression of the miRNAs would enhance HSV-1-induced KSHV replication. We inhibited the function of miR-498 and miR-320d using their respective locked nucleic acid (LNA) miRNA suppressors followed verifying the expression of both miRNAs by RT-qPCR (Fig. S4). As shown in Fig. 3A, the expression of RTA was markedly increased in HSV-1-infected BCBL-1 cells following the suppression of either miR-498 or miR-320d. ORF65-positive cells were also increased 1.68- and 1.44-fold, respectively, in BCBL-1 cells (Fig. 3B and 3C). The expression of ORF57, ORF59 and K9 mRNA transcripts was also enhanced (Fig. 3D). Furthermore, consistent with the increased expression of KSHV lytic genes, suppression of either miR-498 or miR-320d increased the production of viral particles by 6.26- and 4.80-fold, respectively (Fig. 3E). In addition, to determine whether transfection of LNA-498 or LNA-320d induced spontaneous reactivation in BCBL-1 cells, we detected the copy number of KSHV progeny virions in the supernatant of BCBL-1 cells transfectd with LNA-498, LNA-320d or LNA scrambled control alone for 12 h. As expected, transfection of LNA-498 or LNA-320d did induce spontaneous reactivation in BCBL-1 cells (Fig. S5). These results suggest that HSV-1 reactivates KSHV replication by downregulating miR-498 and miR-320d, both of which directly target RTA.

### Both MiR-498 and MiR-320d Regulate KSHV Replication in BCBL-1 Cells without HSV-1 Infection

**Figure 4:**
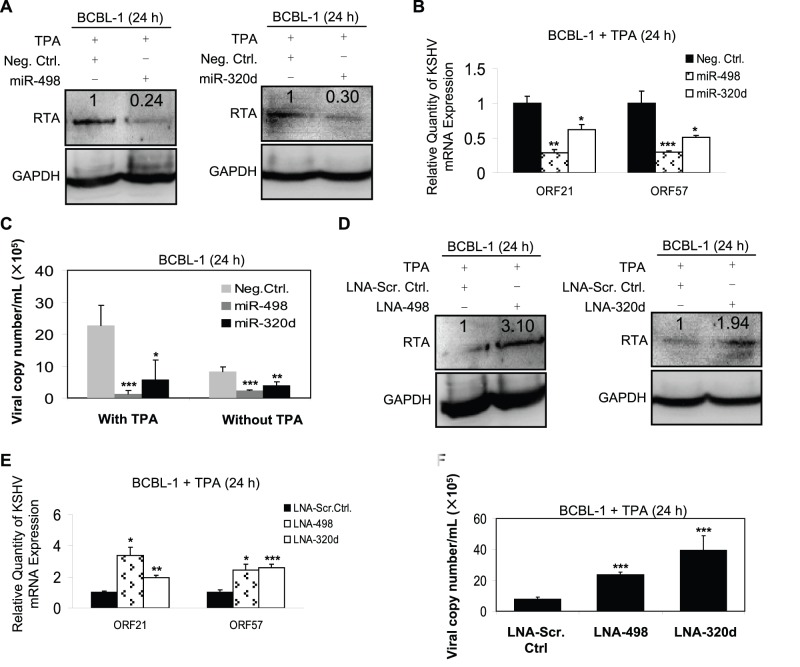
Either miR-498 or miR-320d regulates KSHV replication in BCBL-1 cells without HSV-1 infection. (**A**)**. Transfection of miR-498 or miR-320d mimic inhibited KSHV RTA expression in TPA-stimulated BCBL-1 cells.** Western blot was used to detect the expression of RTA in BCBL-1 cells which were transfected with miR-498, miR-320d mimics or Neg. Ctrl. for 12 h, and stimulated with TPA for another 24 h. The relative level of RTA was determined by quantitative densitometry. Numbers labeled above the RTA band were the relative intensities of the bands compared to GAPDH. The relative level of RTA in the TPA+Neg. Ctrl. group was considered to be 1 for comparison. (**B**)**. Transfection of miR-498 or miR-320d mimic inhibited KSHV ORF21/57 mRNA expression in TPA-stimulated BCBL-1 cells.** ORF21 and ORF57 mRNA in BCBL-1 cells treated as in A was quantitated by RT-qPCR. *P<0.05, **P<0.01 and ***P<0.001 for Student’s t-test versus TPA+Neg. Ctrl. group. (**C**)**. Transfection of miR-498 or miR-320d mimic inhibited KSHV progeny virions release in TPA-stimulated or unstimulated BCBL-1 cells.** Real-time DNA-PCR was used to detect the viral genome copy number in the supernatant of BCBL-1 cells, which were transfected with miR-498, miR-320d mimics or Neg. Ctrl. for 12 h, and treated with TPA for another 24 h (left columns) or without TPA (right columns). *P<0.05, **P<0.01 and ***P<0.001 for Student’s t-test versus Neg. Ctrl. group. (**D**)**. Transfection of LNA-498 or LNA-320d enhanced KSHV RTA expression in TPA-stimulated BCBL-1 cells.** Western blot was used to detect the expression of RTA in BCBL-1 cells transfected with LNA-498, LNA-320d or LNA-Scr. Ctrl. for 12 h, and stimulated with TPA for another 24 h. The relative level of RTA was determined by quantitative densitometry. Numbers labeled above the RTA band were the relative intensities of the bands compared to GAPDH. The relative level of RTA in the TPA+LNA-Scr. Ctrl. group was considered to be 1 for comparison. (**E**)**. Transfection of LNA-498 or LNA-320d enhanced KSHV ORF21/57 mRNA expression in TPA-stimulated BCBL-1 cells.** ORF21 and ORF57 mRNA in BCBL-1 cells treated as in D was quantitated by RT-qPCR. *P<0.05, **P<0.01 and ***P<0.001 for Student’s t-test versus TPA+LNA-Scr. Ctrl. group**.** (**F**)**. Transfection of LNA-498 or LNA-320d enhanced KSHV progeny virions release in TPA-stimulated BCBL-1 cells.** Real-time DNA-PCR was used to detect the viral genome copy number in the supernatant of BCBL-1 cells treated as in D. ***P<0.001 for Student’s t-test versus TPA+LNA-Scr. Ctrl. group.

To explore whether miR-498 or miR-320d alone, without HSV-1 infection, regulated KSHV replication, mimics or LNA of these two miRNAs were transfected into 12-*O*-tetradecanoylphorbol-13-acetate (TPA)-stimulated BCBL-1 cells (TPA was used to amplify the signal, so it became easier to detect gene expression). Transfection of miR-498 or miR-320d mimic without HSV-1 infection not only inhibited the expression of KSHV RTA protein and two KSHV lytic transcripts ORF21/57, but also decreased the copy number of KSHV progeny virions (Fig. 4A–C). Meanwhile, the mutant mimics failed to inhibit the release of KSHV progeny virions induced by TPA (Fig. S6). Expected results were also observed when TPA-stimulated BCBL-1 cells were transfected with miR-498 or miR-320d LNA. As shown in Fig. 4D–F, the expression of RTA protein and ORF21/57 mRNA transcripts was markedly increased, and the production of viral particles was also increased. Notably, regardless of TPA induction, miR-498 and miR-320d had the ability to inhibit KSHV replication reflected from the copy number of KSHV progeny virions (Fig. 4C). Taken together, these data suggest that miR-498 and miR-320d may play an important role in regulating KSHV replication.

### Gene Ontology (GO) and Pathway Analysis Reveal the Roles of Up- and Downregulated MiRNAs

**Figure 5:**
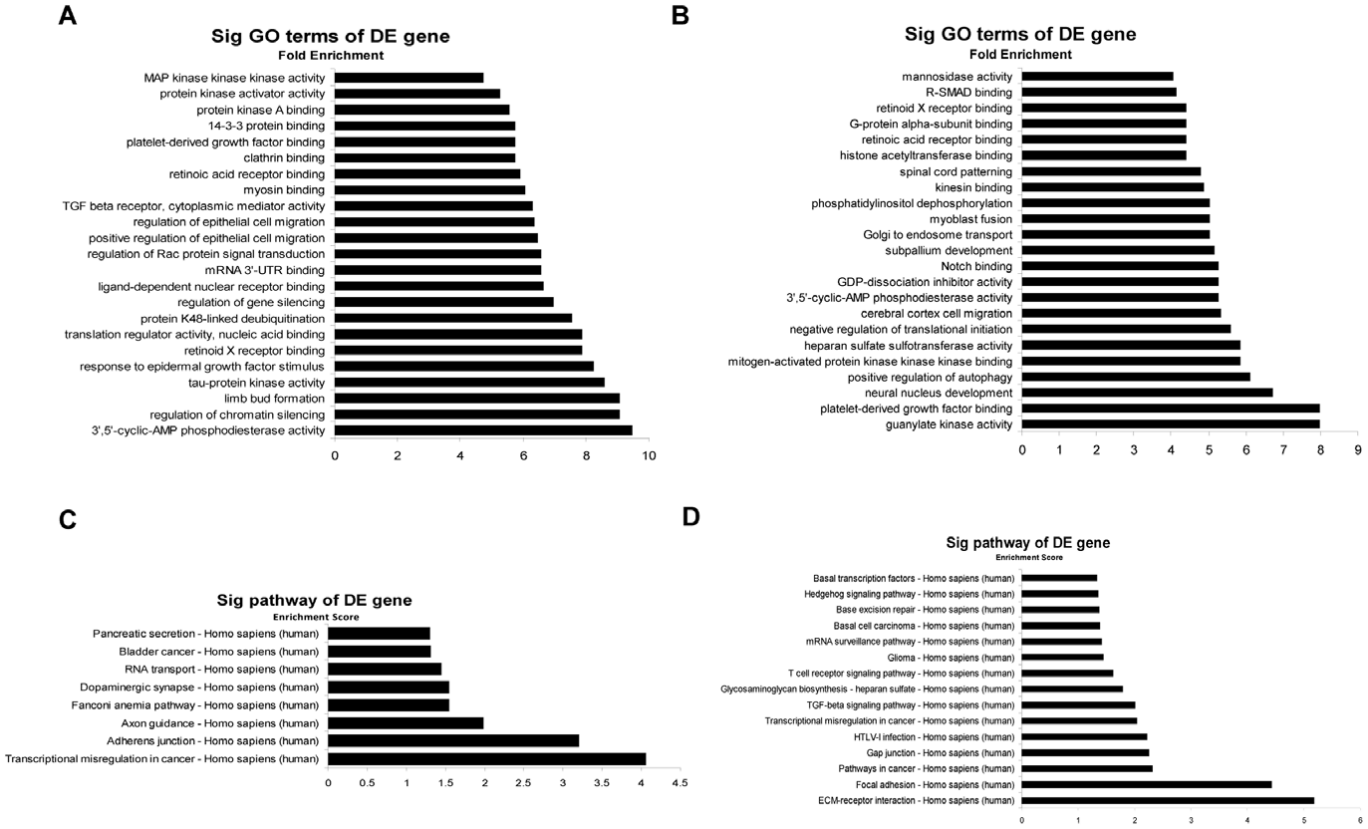
Bioinformatics GO and pathway analysis based on miRNAs target genes. (**A–B**)**. GO analysis of the predicted target genes of miRNAs.** Only the top 23 significant GO terms for differentially up-(A) or downregulated (B) miRNAs were listed. The vertical axis was GO category and the horizontal axis was fold enrichment, which equaled (Count/Pop.Hits)/(List.Total/Pop.Total) and represented the significant level of GOs [Count: the number of differentially expressed (DE) genes associated with the listed gene ontology term; Pop.Hits: the number of background population genes associated with the listed gene ontology term; List.Total: the total number of DE genes; Pop.Total: the total number of background population genes**].** (**C–D**)**. KEGG pathway analysis based on miRNAs target genes.** The top significant pathways targeted by differentially up-(C) or downregulated (D) miRNAs were listed. The vertical axis was pathway category, the horizontal axis was enrichment score, which equaled [-log10(P value)] and represented the significant level of pathways.

As shown in Table 1, 109 miRNAs were downregulated and 75 miRNAs were upregulated after BCBL-1 cells were infected by HSV-1 for 24 h. To uncover functional roles of these differentially expressed miRNAs, GO category and KEGG (Kyoto Encyclopedia of Genes and Genomes) pathway analysis of their target pool were applied. We noticed that the above miRNA targets were especially enriched for proteins with roles in protein binding, enzyme activity and biological regulation (Fig. 5A and 5B). Another functional analysis of up- and downregulated miRNAs by KEGG annotation revealed that some signal transduction pathways, such as transcriptional misregulation in cancer, pathway in cancer and transforming growth factor (TGF)-β signaling pathway, were regulated by the identified miRNAs (Fig. 5C and 5D). These results provide insights into the functions of these miRNAs and may be helpful in identifying the other important miRNAs that are involved in HSV-1-induced KSHV replication and the development of KS.

## Discussion

MiRNAs are a class of endogenous, small noncoding RNAs with important post-transcriptional regulatory functions by base pairing to the 3′UTR of target mRNAs, and work by direct cleavage of the target mRNAs or by inhibition of protein synthesis. The influence of miRNAs on the host–pathogen environment is largely unknown and under intensive investigation. Whether produced by the pathogen or by the host cell, these miRNAs sculpt the intracellular landscape, because their activity ultimately affects levels of target proteins.

It is well known that KSHV molecular switch RTA is an IE protein and initiation of KSHV lytic replication is mediated by RTA [28]. Our results showed that HSV-1 induced KSHV lytic replication by downregulation of miR-498 and miR-320d, which upregulated RTA expression. In fact, some herpesviruses-encoded miRNAs (thus far, nearly all herpesviruses have been found to encode their own miRNAs) were involved in viral life cycle control by regulating the expression of viral IE genes. HSV-1-encoded miR-H2-3p and miR-H6 facilitated establishment and maintenance of viral latency by targeting viral IE transactivators ICP0 and ICP4, respectively [48]. Similar results have been observed in HSV-2, in which miR-II and miR-III contributed to viral latency through silencing ICP34.5 and ICP0, respectively [49]. In HCMV, miR-UL112-1 inhibited viral lytic replication by inhibiting IE72 [50]. Studies in KSHV showed that several KSHV-encoded miRNAs controlled viral latency by affecting RTA expression, either directly or indirectly. MiR-K5, miR-K7-5p and miR-K9-5p were found to target RTA directly and thereby antagonized KSHV reactivation [35], [51], [52]. A study by Lu and colleagues has revealed that miR-K3 reduced RTA mRNA levels by targeting nuclear factor (NF) I/B [39]. Lei et al. found that IκBα (inhibitor of NF-κB) was the target of miR-K1, and inhibition of IκB leaded to NF-κB activation, which suppressed RTA to facilitate viral latency [36]. All these findings clearly suggest that viral miRNAs are used by herpesviruses to target IE genes to regulate viral life cycle. Therefore, it is not surprising that cellular miRNAs are capable of regulating KSHV replication by targeting RTA.

The host- or virus-encoded miRNAs, and their target genes, together form novel regulatory networks between the host and virus. Here, we explored the effect of host-encoded miRNAs on KSHV replication and found that miR-498 and miR-320d negatively regulated KSHV lytic replication. MiR-498 and miR-320 have been considered to be relevant to carcinogenesis. MiR-498 and miR-320 were highly expressed in retinoblastoma and in malignantly transformed 16HBE-T human bronchial epithelial cells [53], [54]. MiR-498 and miR-320 have been proved to be correlated with the probability of recurrence-free survival in stage II colon cancer patients [55]. A recent study indicated that downregulation of miR-320 and upregulation of one of its direct targets, ETS2, were critical events in phosphatase and tensin homolog deleted on chromosome ten (PTEN)-deleted stromal fibroblasts responsible for promoting tumor angiogenesis and tumor cell invasion [56]. Another study also showed that downregulation of miR-320 in HCV-infected cells might play some role in hepatocarcinogenesis, because miR-320 had a tendency to suppress genes related to carcinogenesis [57]. MiR-320d [the human genome contains eight annotated miR-320 genes (miR-320a, miR-320b-1/2, miR-320c-1/2, miR-320d-1/2, miR-320e) that encode five mature miR variants (miR-320a/b/c/d/e)] was found to be downregulated in human colon cancer stem cells (CSCs) and might play important roles in maintaining stemness of colon CSCs [58]. Furthermore, miR-320 was reported to induce G1 arrest and suppress cell proliferation by targeting CDK6, CD71, insulin-like growth factor (IGF)-1 and induce apoptosis by suppressing Bcl-2 and Mcl-1 [59]–[62]. Besides association with carcinogenesis, miR-498 appeared to be involved in the development of certain diseases. In rheumatoid arthritis patients, decreased expression of miR-498 has been found in CD4^+^ T cells from synovial fluid or peripheral blood [63]. Downregulation of miR-498 was also found in the nasal mucosa of allergic rhinitis patients [64]. In addition, miR-498 was has recently been reported to play a possible role in translational repression of 11β-hydroxysteroid dehydrogenase type 2 mRNA in BeWo cells (human placental cell line) subjected to amino acid deficiency [65]. However, in our study, miR-498 and miR-320d played an important role in regulating KSHV replication.

HSV-1 infection of BCBL-1 cells altered the expression of a number of miRNAs, we have digged out the two key miRNAs, miR-498 and miR-320d, among the downregulated miRNAs, whether several of upregulated miRNAs mediated HSV-1-induced KSHV replication by targeting negative regulators of RTA are needed to be validated. The results of GO enrichment and KEGG pathway analysis would further delineate the influences of the other important cellular miRNAs involved in HSV-1-induced KSHV replication and the development of KSHV-related malignancies. The information from KEGG annotation showed that cell communication (focal adhesion, gap junction and adherens junction), genetic information processing (mRNA surveillance pathway, base excision repair, basal transcription factors, fanconi anemia pathway and RNA transport), signal transduction and interaction (ECM-receptor interaction, TGF-β signaling pathway and Hedgehog signaling pathway) were numerous among the significantly enriched pathways. Remarkably, we noticed that downregulated miRNA targets were especially enriched for MAPKKK (mitogen-activated protein kinase kinase kinase) binding, which interacted selectively and non-covalently with a MAPKKK, or phosphorylated a MAPKK (mitogen-activated protein kinase kinase). Recently, we have reported that PTEN/phosphatidylinositol 3-kinase (PI3K)/AKT (also called protein kinase B, PKB)/glycogen synthase kinase-3β (GSK-3β) and extracellular signal-regulated protein kinase (ERK) mitogen-activated protein kinase (MAPK) signal pathways contributed to HSV-1-induced KSHV replication [10]. Therefore, further experiments will determine whether two pathways are targeted by these miRNAs, especially whether members of MAPK signal pathway are targeted by the essential miRNAs among those regulated miRNAs.

In conclusion, we provided evidence for interplay between cellular miRNAs and KSHV. HSV-1 infection of BCBL-1 cells reduced the expression levels of miR-498/miR-320d and inversely enhanced target RTA levels, which switched KSHV lytic replication. The identification of miRNAs that regulate KSHV replication and their targets not only enable us to understand better the molecular basis of KSHV pathogenesis, but also enable us to develop better therapeutic strategies.

## Materials and Methods

### Cells Culture and Virus Infection

BCBL-1 and BC-3 cells (KSHV-positive and EBV-negative PEL cell lines) were obtained through the AIDS Research and Reference Reagent Program, National Institutes of Health (Bethesda, MD) and maintained in RPMI-1640 containing 10% fetal bovine serum (FBS), 2 mmol/l L-glutamine, 100 U/ml penicillin, and 100 µg/ml streptomycin at 37°C in a humidified, 5% CO2 atmosphere. Vero cells (African green monkey kidney fibroblasts) and 293T cells were obtained from American Type Culture Collection (ATCC, Rockville, MD) and grown in Dulbecco’s modified Eagle’s medium (DMEM) +10% FBS. HSV-1 (McKrae strain) propagated in Vero cells and viral titers were also determined in Vero cells. The supernatant from normal Vero cells culture was used as a control (Mock). HSV-1 infection of BCBL-1 cells was described previously [9].

### MiRNA Microarray Analysis

Total RNA from BCBL-1 cells infected with HSV-1 or Mock for 24 h was isolated with Trizol reagent (Invitrogen, Carlsbad, CA) and miRNA fraction was purified further using a mirVana TM miRNA isolation kit (Ambion, Austin, TX). The isolated miRNAs from the two parallel infected-cells were then labeled with Hy3 using the miRCURYTM Array Labelling kit (Exiqon, Vedbaek, Denmark) and hybridized respectively on a miRCURY TM LNA microRNA Array (v 8.0, Exiqon). Microarray images were acquired using a Genepix 4000B scanner (Axon Instruments, Union City, CA) and processed and analyzed with Genepix Pro 6.0 software (Axon Instruments).

### MiRNA Mimics and Suppressors

Synthetic miR-498 and miR-320d mimics and miRNA mimic negative control (Neg. Ctrl.) were obtained from Shanghai GenePharma Company (Shanghai, China). The sequences of miRNA mimics and their stem-loops were shown in Table 2. To simulate functions of miRNAs, 100 nmol/L of mimics were transfected into cells. MiRCURY LNA miRNA suppressors and the scrambled control (Scr. Ctrl.) were obtained from Exiqon (Vedbaek, Denmark). To suppress functions of miRNAs, 75 nmol/L of LNA oligonucleotides were transfected into cells.

**Table 2:** The sequences of the negative control mimic, the mature miR-498 and miR-320d mimics and their stem-loops.

MiRNA mimic and stem-loop			Sequence
hsa-miRNA negativecontrol mimic	5′ to 3′	Sense	UUCUCCGAACGUGUCACGUTT
hsa-miR-498mimic	5′ to 3′	Sense	UUUCAAGCCAGGGGGCGUUUUUC
hsa-miR-498stem-loop	5′ to 3′	Sense	AACCCUCCUUGGGAAGUGAAGCUCAGGCUGUGAUUUCAAGCCAGGGGGCGUUUUUCUAUAACUGGAUGAAAAGCACCUCCAGAGCUUGAAGCUCACAGUUUGAGAGCAAUCGUCUAAGGAAGUU
hsa-miR-320dmimic	5′ to 3′	Sense	AAAAGCUGGGUUGAGAGGA
hsa-miR-320dstem-loop	5′ to 3′	Sense	UUCUCGUCCCAGUUCUUCCCAAAGUUGAGAAAAGCUGGGUUGAGAGGA

### Plasmids, Transfection and Luciferase Reporter Assay

The construct pGL3-Luc-RTA 3′UTR (RTA wild-type) and RTA-expressing plasmid pcDNA3.1–3×Flag-RTA-3′UTR were described elsewhere [36]. The plasmid pEGFP-N2 was from CLONTECH Laboratories (Palo Alto, CA). MiR-498 and miR-320d targeting sites were identified with target prediction software RNAhybrid, PITA and FindTar. Mutations of the putative miR-498-binding site S2 from RTA 3′UTR were performed by site-directed mutagenesis using primers 5′-GTT TTC AGA GGA ACG CTT ATG CAC-3′ and 5′-TTT CTA TGT AGT CGC CTC TTG GAT-3′. Mutations of the putative miR-320d-binding site S1 from RTA 3′UTR were performed also by site-directed mutagenesis using primers 5′-ACC ATG ATT TAC GGA CAA TAC TGA C-3′ and 5′-TGC CCG TTG AGG CTT AGA TCT TC-3′. All DNA and miRNA transfection experiments were performed with Lipofectamine 2000 (Invitrogen) following the manufacturer’s instructions. After 24 h transfection, cells were harvested for luciferase assay. Renilla vector pRL-TK (Promega, Madison, WI) was used as an internal control and relative luciferase activity was assayed using Promega dual-luciferase reporter assay system.

### Antibodies and Reagents

Anti-KSHV ORF65 mouse monoclonal antibody (mAb) was kindly provided by S-J Gao (University of Southern California, CA, USA) [66]. Anti-KSHV RTA antibody was generated by immunization of rabbits with ORF50 peptide (amino acids 667–691) [67]. Anti-Flag M2 rabbit mAb and anti-GFP rabbit antibody were from Cell Signaling Technology (Beverly, MA, USA) and Beyotime Institute of Biotechnology (China), respectively. Mouse mAbs against α-tubulin/GAPDH and horseradish peroxidase (HRP)-conjugated goat anti-mouse/rabbit IgG were purchased from Santa Cruz Biotechnology (Santa Cruz, CA, USA). TPA (20 ng/ml) was obtained from Sigma (St. Louis, MO, USA).

### Western Blot Analysis

Western blot was performed as described previously [10]. Differences in protein expression were determined by densitometry analysis using Scion Image software (Scion Corporation, Frederick, MD). Data shown were repeated at least three times to confirm results.

### RT-qPCR

Total RNA was isolated from cells by using Trizol reagent (Invitrogen). RT-qPCR was performed using SYBR *Premix Ex Taq*™ Kit (TaKaRa Biotechnology Co.Ltd., Dalian, China) according to the manufacturer’s instructions. Bulge-Loop™ miRNA RT-qPCR primer sets (one reverse transcription primer and a pair of qPCR primers for each set) specific for miR-498 and miR-320d were designed by RiboBio (Guangzhou, China). The sequences of specific primers of RT-qPCR for KSHV K9 and ORF21/57/59 genes were listed in Table 3. All the reactions were run in triplicates.

**Table 3:** Primers used for RT-qPCR detection of KSHV genes (F, Forward; R, Reverse).

Target	Primer	Target	Primer
ORF21	F: 5′-CGT AGC CGA CGC GGA TAA-3′R: 5′-TGC CTG TAG ATT TCG GTC CAC-3′	K9	F: 5′-GTC TCT GCG CCA TTC AAA AC-3′R: 5′-CCG GAC ACG ACA ACT AAG AA-3′
ORF57	F: 5′-TGG CGA GGT CAA GCT TAA CTT C-3′R: 5′-CCC CTG GCC TGT AGT ATT CCA-3′	β-actin	F: 5′-TTG CCG ACA GGA TGC AGA AGG A-3′R: 5′-AGG TGG ACA GCG AGG CCA GGA T-3′
ORF59	F: 5′-TTG GCA CTC CAA CGA AAT ATT AGA A-3′R: 5′-CGG GAA CCT TTT GCG AAG A-3′		

### Real-time DNA-PCR, IFA, GO and Pathway Analysis

Both the experiments of real-time DNA-PCR for viral copy number and IFA were performed as previously described [10]. GO and pathway analysis was carried out as described elsewhere [68], [69]. Fisher’s exact test was used to classify GO category and KEGG pathway. *P*-value <0.05 was used as a threshold to select significant GO categories and KEGG pathways.

## Supporting Information

Figure S1 HSV-1 infection of BCBL-1 cells downregulates miR-498 and miR-320d as early as 3 h post-infection. MiR-498 (**A**) and miR-320d (**B**) expression in BCBL-1 cells infected with HSV-1 or Mock for 0, 3, 6 and 12 h was quantitated by RT-qPCR. Relative quantities of miRNAs expression were represented as 2^−ΔΔCt^ on the y axis. ***P<0.001 for Student’s t-test versus Mock group.
(EPS)

Figure S2 Overexpression of mimics of miR-498 and miR-320d increases their own expression. RT-qPCR was used to detect the expression of miR-498 (**A**) and miR-320d (**B**) in BCBL-1 cells transfected with miR-498, miR-320d mimics or negative control nucleotide of miRNA (**Neg. Ctrl.**) for 12 h, and infected with HSV-1 or Mock for another 24 h. **P<0.01 for Student’s t-test versus Mock+Neg. Ctrl. group. *n.s.*, not significant for Student’s t-test versus Mock+Neg. Ctrl. Group.
(EPS)

Figure S3 Transfection of miR-498 or miR-320d mimics inhibits HSV-1-induced KSHV progeny virions release in BC-3 cells. Real-time DNA-PCR was used to detect the viral genome copy number in the supernatant of BC-3 cells, which were transfected with miR-498, miR-320d mimics or Neg. Ctrl. for 12 h, and infected with HSV-1 or Mock for another 24 h. *P<0.05 for Student’s t-test versus HSV-1+ Neg. Ctrl. group.
(EPS)

Figure S4 Inhibition of miR-498 and miR-320d decreases their own expression. RT-qPCR was used to detect the expression of miR-498 (**A**) and miR-320d (**B**) in BCBL-1 cells transfected with LNA-498, LNA-320d or LNA scrambled control (**LNA-Scr. Ctrl.**) for 12 h, and infected with HSV-1 or Mock for another 24 h. *P<0.05, **P<0.01, and ***P<0.001 for Student’s t-test versus Mock+LNA-Scr. Ctrl. group.
(EPS)

Figure S5 Transfection of LNA-498 or LNA-320d induces KSHV spontaneous reactivation in BCBL-1 cells. Real-time DNA-PCR was used to detect the viral genome copy number in the supernatant of BCBL-1 cells transfected with LNA-498, LNA-320d or LNA-Scr. Ctrl. for 12 h. *P<0.05 for Student’s t-test versus LNA-Scr. Ctrl. group.
(EPS)

Figure S6 Transfection of miR-498 or miR-320d mimics inhibits KSHV progeny virions release in TPA-stimulated BCBL-1 cells. Real-time DNA-PCR was used to detect the viral genome copy number in the supernatant of BCBL-1 cells transfected with Neg. Ctrl. (**Neg. Ctrl.**), mimic of miR-498 (**miR-498**), mimic of miR-320d (**miR-320d**), mutant mimic of miR-498 (**mut miR-498**), or mutant mimic of miR-320d (**mut miR-320d**) for 12 h, and stimulated with TPA for another 24 h. **P<0.01 for Student’s t-test versus Neg. Ctrl. group. *n.s.*, not significant for Student’s t-test versus Neg. Ctrl. Group.
(EPS)
